# Greenhouse gas budget of a poplar bioenergy plantation in Belgium: CO_2_ uptake outweighs CH_4_ and N_2_O emissions

**DOI:** 10.1111/gcbb.12648

**Published:** 2019-10-06

**Authors:** Joanna A. Horemans, Nicola Arriga, Reinhart Ceulemans

**Affiliations:** ^1^ Department of Biology Research Center of Excellence PLECO University of Antwerp Wilrijk Belgium; ^2^ CzechGlobe Academy of Sciences Brno Czech Republic; ^3^ Slovenian Forestry Institute Ljubljana Slovenia

**Keywords:** bioenergy, CO_2_ uptake outweighs CH_4_ and N_2_O emissions, greenhouse gas balance, plantation establishment, *Populus*, short‐rotation coppice

## Abstract

Biomass from short‐rotation coppice (SRC) of woody perennials is being increasingly used as a bioenergy source to replace fossil fuels, but accurate assessments of the long‐term greenhouse gas (GHG) balance of SRC are lacking. To evaluate its mitigation potential, we monitored the GHG balance of a poplar (*Populus*) SRC in Flanders, Belgium, over 7 years comprising three rotations (i.e., two 2 year rotations and one 3 year rotation). In the beginning—that is, during the establishment year and during each year immediately following coppicing—the SRC plantation was a net source of GHGs. Later on—that is, during each second or third year after coppicing—the site shifted to a net sink. From the sixth year onward, there was a net cumulative GHG uptake reaching −35.8 Mg CO_2_ eq/ha during the seventh year. Over the three rotations, the total CO_2_ uptake was −51.2 Mg CO_2_/ha, while the emissions of CH_4_ and N_2_O amounted to 8.9 and 6.5 Mg CO_2_ eq/ha, respectively. As the site was non‐fertilized, non‐irrigated, and only occasionally flooded, CO_2_ fluxes dominated the GHG budget. Soil disturbance after land conversion and after coppicing were the main drivers for CO_2_ losses. One single N_2_O pulse shortly after SRC establishment contributed significantly to the N_2_O release. The results prove the potential of SRC biomass plantations to reduce GHG emissions and demonstrate that, for the poplar plantation under study, the high CO_2_ uptake outweighs the emissions of non‐CO_2_ greenhouse gases.

## Graphical abstract

Before woody biomass can be considered as a potential alternative for fossil fuels, we need to know whether a biomass plantation is a net source or a net sink of the three most important greenhouse gases (CO_2_, N_2_O, and CH_4_). The operational poplar short‐rotation coppice plantation in Flanders (Belgium) was a net greenhouse gas emitter shortly after the establishment, but it turned into a net sink after approximately 5 years. The CO_2_ flux dominated the greenhouse gas balance and a total of 36 Mg CO_2_ eq/ha was sequestered after 7 years of observation (three rotation cycles).

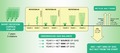

## 1 INTRODUCTION

Reaching the renewable energy targets of the European Commission (EU, 2018) and the United States (US DOE, 2016) requires a mix of energy sources. Among the various renewable energy sources, energy from biomass from short‐rotation coppice (SRC) with fast‐growing trees, as poplar and willow, is a promising option for the production of electric and thermal energy. SRCs are characterized by high yields with average dry mass production rates between 10 and 15 Mg ha^−1^ year^−1^ (Di Matteo, Sperandio, & Verani, 2012; Labrecque & Teodorescu, 2005; Laureysens, Bogaert, Blust, & Ceulemans, 2004; Sixto et al., 2015; Van de Walle, Camp, Casteele, Verheyen, & Lemeur, 2007) and maxima up to 25 Mg ha^−1^ year^−1^ under optimal environmental conditions (Ceulemans et al., 1992; Liberloo et al., 2006). When SRC is established on former cropland, the less intensive tillage and the recurring soil enrichment by dead plant material after coppice can lead to an increase in soil organic carbon (SOC) storage (Berhongaray, Verlinden, Broeckx, Janssens, & Ceulemans, 2017; Don et al., 2012; Grigal & Berguson, 1998; Smith, 2004), but not necessarily (Pacaldo, Volk, & Briggs, 2013; Walter, Don, & Flessa, 2015). The effect of SRC establishment is not always visible immediately after land conversion (Arevalo, Bhatti, Chang, & Sidders, 2011; Njakou Djomo et al., 2013) and depends on plantation age (Hansen, 1993), on the former land use, as well as on soil texture, structure, and acidity (Harris, Spake, & Taylor, 2015). An advantage of the conversion of cropland to SRC is the lower nitrogen input requirement which reduces the emission of N_2_O to the atmosphere and improves water quality (Whitaker et al., 2017).

Although biomass from SRC might be a valuable option to partially replace fossil fuels, we lack knowledge of the greenhouse gas (GHG) balance associated with the operation of perennial SRC (Crutzen, Mosier, Smith, & Winiwarter, 2008; Díaz‐Pinés et al., 2016; Palmer, Forrester, Rothstein, & Mladenoff, 2014). Previous life cycle analyses of SRC plantations combined field measurements and modeling (Schweier et al., 2017), but ecosystem GHG fluxes have seldomly been quantified over multiple rotations of SRC on‐site (Gelfand et al., 2013; Harris et al., 2015). Monitoring GHG emissions after land conversion to SRC over only one rotation provides a distorted picture of reality because the largest impact occurs shortly after the land‐use change (see e.g., Nikiema, Rothstein, & Miller, 2012; Palmer et al., 2014; Walter et al., 2015; Zenone et al., 2016). Most previous studies only measured the exchanges of CO_2_ neglecting important non‐CO_2_ GHGs as nitrous oxide (N_2_O) and methane (CH_4_). Compared to CO_2_, the absolute fluxes of CH_4_ and N_2_O are smaller, but their global warming potential is 25, respectively, 298 times larger than that of CO_2_ (Forster et al., 2007). In natural ecosystems, CH_4_ is mostly emitted from swamps (Conrad, 1996), where anaerobic conditions stimulate its biological formation. N_2_O is formed during microbial nitrification and denitrification with the emission of N_2_O depending on the availability of ${\text{NO}}_{3}^{-}$ (Palmer et al., 2014). The environmental drivers of N_2_O and CH_4_ emissions are largely unknown, and observations of the fluxes of these gases produce varying results (Harris et al., 2015). Former land use, site‐specific soil properties and climate conditions influence GHG emissions from SRCs (Field, Marx, Easter, Adler, & Paustian, 2016; Whitaker et al., 2017). Site management, that is, the use of fertilizer, irrigation, and length of the rotation period, also influences the GHG balance (Carter et al., 2012; Díaz‐Pinés et al., 2016).

The goals of the present study were to monitor the net (atmosphere to plantation) fluxes of the three most important GHGs, and to reconstruct the GHG balance of an operational SRC plantation. We hypothesize that the SRC plantation is a net sink of GHGs and that this sink increases with time.

## 2 MATERIALS AND METHODS

### 2.1 Study area

The operational poplar SRC plantation covers an area of 14.5 ha and is located in Lochristi, East Flanders, Belgium (51°06′44″N, 3°51′02″E, 6.25 m a.s.l.). It is being used to produce woody biomass for the production of renewable electricity and “green” heat. The long‐term average annual and growing season temperatures at the site are 9.5 and 13.7°C, respectively. Average annual and growing season precipitation is 726 and 433 mm, respectively (Broeckx, Verlinden, & Ceulemans, 2012). On April 7–10, 2010, hardwood cuttings of 12 commercially available poplar genotypes and three willow genotypes were planted at a density of 8,000 cuttings/ha in a double row planting scheme with alternating distances of 0.75 and 1.5 m between the rows and 1.1 m between trees within rows (Broeckx et al., 2012). Before the SRC plantation was established, and for at least 20 years, 62% of the area was cultivated with regularly fertilized (200–300 kg ha^−1^ year^−1^ of fertilizer) agricultural crops such as ryegrass, sugar beet, wheat, potatoes, and most recently maize. The remaining 38% of the area was intensively grazed pasture. The 2010 soil analysis showed on average 84.7% sand and 11.3% clay (Verlinden, Broeckx, Wei, & Ceulemans, 2013). Since the establishment of the SRC in 2010, neither fertilization nor irrigation has been applied. During the first month after land conversion to SRC and after each coppicing, conventional manual and chemical weed control (Ledin & Willebrand, 1996) was performed.

**Figure 1:**
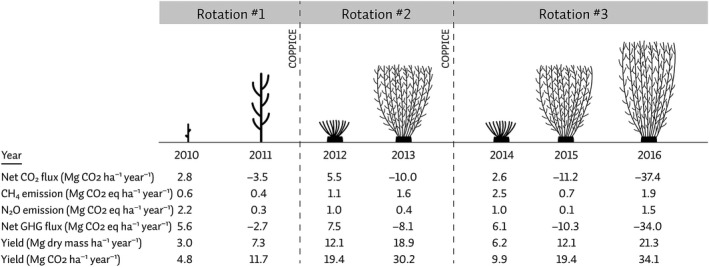
Schematic representation of the vegetation structure during the three rotations of the short‐rotation coppice plantation, together with the net cumulative CO_2_, CH_4_, N_2_O and total net greenhouse gas (GHG) fluxes. Fluxes of CH_4_ and N_2_O fluxes were expressed in CO_2_ equivalents. Negative flux indicates a net uptake (sink); positive flux indicates as net emission (source). Yield values (after Vanbeveren & Ceulemans, 2018) were expressed in dry biomass production per year as well as converted in CO_2_ units

For the first two rotations, the plantation was coppiced every 2 years (Figure 1) with the first harvest taking place on February 2–3, 2012 and the second harvest on February 16–17, 2014. The third rotation was extended to 3 years with the most recent coppice from February 28 to March 1, 2017. At the end of each growing season inventories of shoot diameters at 0.22 m height, the number of shoots per stool and stool mortality were made and used to estimate annual yield (Vanbeveren & Ceulemans, 2018). Above‐ground woody biomass yield values (Figure 1) were obtained from upscaling shoot diameter—dry weight relationships and from the shoot diameter inventories. At harvesting, these relationships were validated with weight measurements of the lorries with harvested biomass (described in Verlinden, Broeckx, & Ceulemans, 2015).

### 2.2 Environmental variables

Air temperature and relative humidity were measured at half‐hourly time steps using Vaisala probes (HMP45C; Vaisala). Soil water content was continuously measured using soil moisture probes (TDR model CS616; Campbell Scientific Inc.) at a depth of 0.2, 0.3, 0.4, 0.6, and 1 m. The water table depth was monitored each half hour using a pressure transducer (PDCR 1830; Campbell Scientific Inc.). Both soil water content and water table depth were measured at five locations, chosen to be representative of the sensed part of the ecosystem. Precipitation data were obtained from the Royal Meteorological Institute at the nearby meteorological station in Zelzate (51°10′53″N, 3°48′33″E, 87.19 m a.s.l.). Occasionally, the site was flooded for 1–2 days following intensive precipitation events during the period 2010–2016. More details about the environmental instrumentation and measurements have been previously published (Zona et al., 2013).

### 2.3 CO_2_, CH_4_, and N_2_O flux measurements and post‐processing

Fluxes of CO_2_, CH_4_, and N_2_O were monitored at half‐hourly resolution from an eddy covariance system. The measurement height for the eddy covariance instruments was adjusted twice per year to track the growth of the canopy, with a minimum measurement height of 5.6 m and a minimum distance from the canopy top of approximately 3.5 m. The footprint of the mast over the research site was maximized by installing it according to the prevailing southwest wind direction (Zona et al., 2013). From April 2010 until February 2014, a sonic anemometer (CSAT3; Campbell Scientific Inc.) was used to measure the three‐dimensional wind speed components. Fluctuations in gas concentrations were measured by a closed‐path fast response infrared gas analyzer for CO_2_ and H_2_O (LI‐7000; LI‐COR Inc.) and by two laser spectrometers for N_2_O and CH_4_ (908‐0014 and DLT‐100; Los Gatos Research Inc., respectively). In February 2014, the above instruments were replaced by a Gill‐HS50 sonic anemometer (Gill Instruments Ltd), an LI‐7200 closed‐path infrared gas analyzer (LI‐COR Inc.) for CO_2_/H_2_O and a single laser spectrometer N_2_O/CH_4_ analyzer (standard rackmount analyzer N2OM1; Los Gatos Research Inc.). All instruments sampled at a frequency of 10 Hz using a data logger (model CR 3000; Campbell Scientific Inc.).

The raw high frequency data were then used to calculate 30 min average fluxes of sensible heat (H), latent heat (LE), CO_2_, CH_4_, and N_2_O using a set of standardized post‐processing calculations and corrections. The most important were: two‐dimensional coordinate rotation to set lateral and vertical mean wind speed to zero; time lag between each scalar and wind speed measurements, estimated through covariance maximization; empirical frequency correction for high‐frequency attenuation and Webb–Pearman–Leuning correction for density fluctuations when needed, that is, when the concentration was not measured as a mixing ratio. Details of these corrections have been provided by Aubinet et al. (2012).

Half‐hourly data were filtered for the entire period with the following criteria: fluxes with a high degree of non‐stationarity and a low level of developed turbulence were excluded; results obtained for wind directions outside the range 50°–250° were also excluded to maximize the representativeness of the measurements collected at the eddy covariance mast; finally, a friction velocity threshold of 0.2 m/s was used for the full dataset. Afterward, net ecosystem exchange, LE, and H were gap‐filled using the marginal distribution sampling methodology (Reichstein et al., 2005). This data processing was achieved using the tool REddyProc provided online by the Max Planck Institute (https://www.bgc-jena.mpg.de/bgi/index.php/Services/REddyProcWeb). For the CH_4_ and N_2_O fluxes, no such tool was available because functional relationships for these fluxes have not yet been described. We, therefore, used an average‐value approach to fill CH_4_ and N_2_O flux data gaps based on the fact that similar conditions were assumed to drive similar fluxes (Mishurov & Kiely, 2011). An averaging window of limited time length (15 days) was used to ensure coherence of environmental and phenological conditions within each gap filling window. This approach is identical to the one previously used for the first two rotations of the site (Zenone et al., 2016).

All post‐processing and gap‐filling methods were consistent with the approaches previously used for the analysis of the first years of the plantation (Carter et al., 2012; Field et al., 2016; Zenone et al., 2016; Zona et al., 2013). All flux data below refer to the measured net exchange fluxes of CO_2_, CH_4_, and N_2_O between the atmosphere and the plantation. Negative fluxes relate to a net uptake from the atmosphere (the plantation is a net sink); positive fluxes relate to a net emission from the plantation to the atmosphere (the plantation is a net source). The absolute coefficient of variation (COV) of the fluxes was calculated as the ratio of the standard deviation over the mean of the absolute values of the half‐hourly flux values over the entire year.

## 3 RESULTS

### 3.1 CO_2_ fluxes and their temporal dynamics

**Figure 2:**
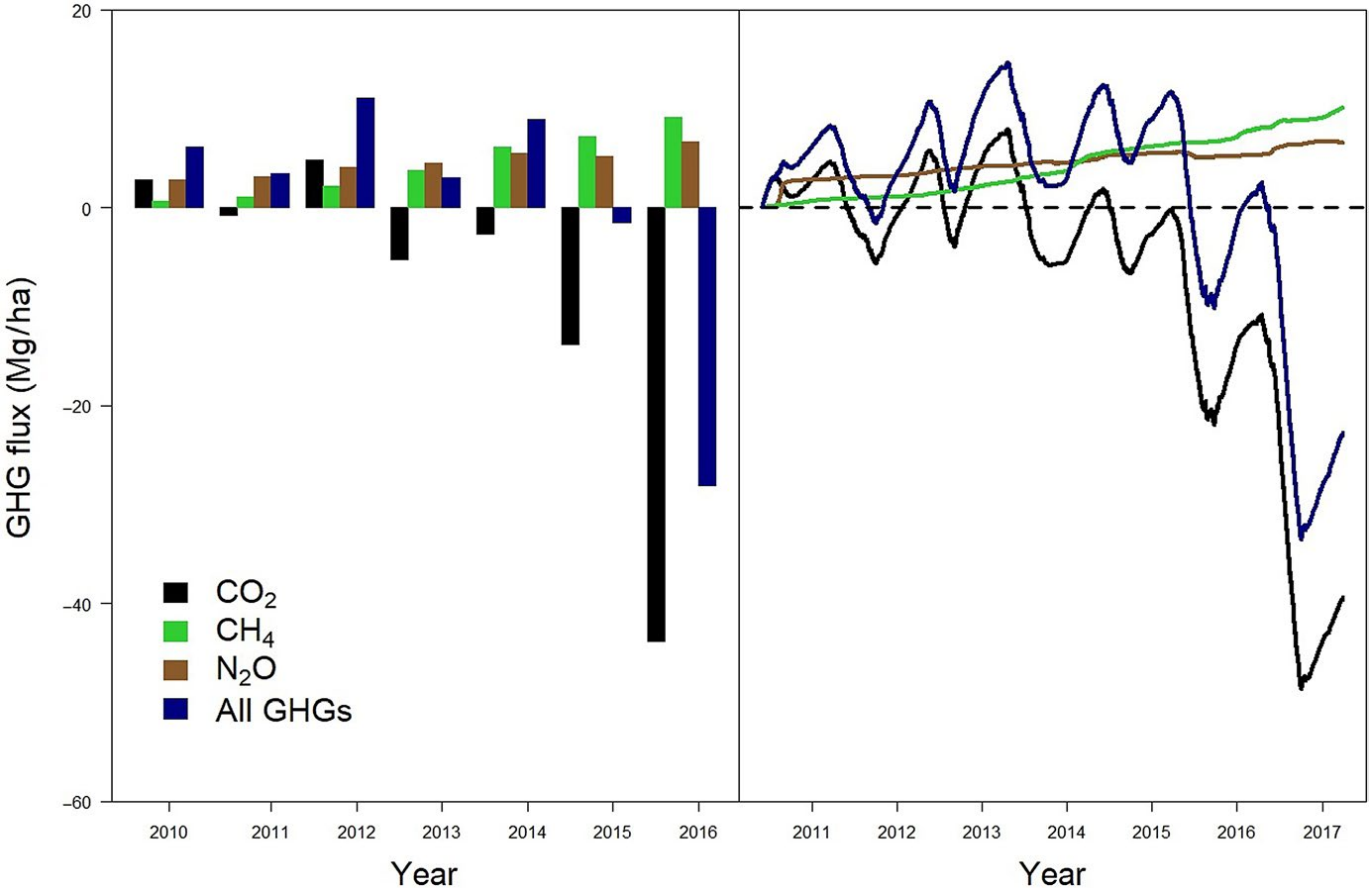
Left panel: Bars represent annual sums of CO_2_, N_2_O, CH_4_ and the total amount of greenhouse gases (in CO_2_ equivalents) for the establishment year (April–December 2010) and for each consequent year. Right panel: Cumulative flux of CO_2_, N_2_O, CH_4_, and the total amount of greenhouse gases (in CO_2_ equivalents). Positive flux values refer to emissions (source); negative fluxes refer to uptake (sink)

After land conversion from cropland and pasture to SRC (in 2010) and after each coppice harvest (2012 and 2014), the plantation was a net source of GHGs (Figures 1 and 2). In each second or third year after coppice (2011, 2013, 2015, and 2016), the plantation shifted into a net sink of GHGs. Moreover, 5 years after the land conversion, the newly established SRC had turned into a net (cumulative) GHG sink (Figure 2). Over the entire duration of the study, the net CO_2_ and GHG fluxes showed the expected temporal dynamics. As expected at this non‐irrigated, non‐flooded, and non‐fertilized site, CO_2_ dominated the GHG balance. Thus, the inter‐annual dynamics of the GHG balance were dominated by the temporal dynamics of the net CO_2_ fluxes (Figure 2). In the second rotation, there was a large difference in the total net flux between both years: a net uptake of −10 Mg ha^−1^ year^−1^ during the second year versus a net emission of +5.5 Mg ha^−1^ year^−1^ during the first year (of the second rotation) which can only be explained by the boost in growth (Vanbeveren et al., 2016).

**Figure 3:**
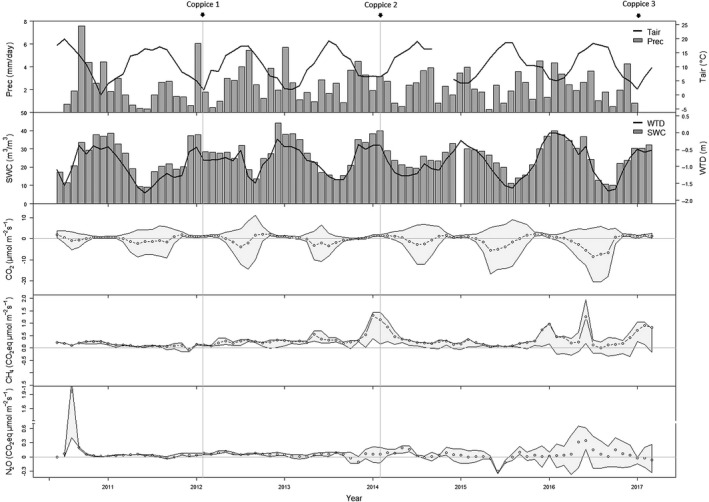
(From top to bottom panels): Monthly mean air temperature (*T*_air_, °C), mean amount of daily precipitation (Prec, mm/day), mean soil water table depth (WTD, m), mean soil water content at 0.2 m soil depth (SWC, m^3^/m^3^), and monthly mean fluxes of CO_2_, CH_4_, and N_2_O (µmol m^−2^ s^−1^ of CO_2_ equivalents) with the 25 and 75 percentile of the distribution of the aggregated half‐hourly values

Respiration of the plantation (i.e., positive net fluxes) increased as the new rotation started regrowing. The coppice harvest also disturbed the soil, and quite some plant residue (branches, shoots) was left on the soil surface, causing an increase in soil respiration and resulting in higher CO_2_ emissions. During the second rotation, the CO_2_ uptake (hence photosynthesis of the plantation) was lower in the last year while during the first and third rotations, the last years showed a higher uptake than during the first year after coppice (Figure 3). Likely environmental variables (see Figure S2a,b) were controlling fluxes beside coppicing, and phenological processes as leaf area development and regrowth after coppice. In the third year of the last rotation, the plantation became an impressive net sink of CO_2_ (−37.4 Mg/ha of CO_2_).

The magnitude of the CO_2_ flux as well as its within‐year variability increased with increasing rotations (Figure 3). The least variable year was 2010 (COV of 1.29) while the year 2013 was the most variable year (COV of 1.78). The sink increased with time, regardless of rotation duration: the ratio's to the second year increased with number of rotations, and were highest for the third year of the third rotation (Table 1). The sink increased from the first to the second and to the third rotation in the same years (first and second year); the sink further increased during the third year, suggesting the adoption of longer rotations (Table 1). The yield in the first 2 years of the third rotation (dry weights of wood of 6.2 and 12.1 Mg ha^−1^ year^−1^, respectively), however, was lower than the yield for the second rotation (12.1 and 18.9 Mg ha^−1^ year^−1^ of wood). It is puzzling that the first 2 years of the third rotation had a larger sink than the first 2 years of the second rotation (Figure 3), but showed a lower yield (Figure 1). Potential explanations for this discrepancy might be (a) changes in SOC could be one explanation, but the “mismatch” seems rather large for this speculation; (b) the root development might be another explanation, but we lack sufficient data to validate this hypothesis. There are furthermore also uncertainties on the yield estimates reported in Figure 1 (from Vanbeveren & Ceulemans, 2018).

**Table 1:** Ratios of net greenhouse gas (GHG) fluxes of the second and third rotations compared to the first and second year's net GHG flux of the first rotation. Ratios were calculated from the values in Figure 1

Year	Ratio to first year	Ratio to second year
Rotation #2	1.34	3.0
Rotation #3	1.09	3.82
Rotation #3, year 3	−6.07	12.59

### 3.2 Non‐CO_2_ fluxes

In comparison with the increasing CO_2_ uptake with time, emissions of N_2_O and CH_4_ were small. CH_4_ emissions remained stable throughout the entire period of the study as shown by the stable slope of the cumulated flux (Figure 2). The absolute COV ranged between 1.08 (2010) and 2.29 (2015). Modest peak CH_4_ emission events were observed in May 2013, at the start of 2014, as well as between November 2015 and May 2016 (Figure 3). Small positive CH_4_ fluxes were also observed from November 2015 onward, with maxima in January and in May 2016. The water table depth and the soil water content at 0.2 m depth both explained a significant part of the monthly variability of CH_4_ fluxes (Figure S3a,b). At the beginning of 2016, average measured water table depths were only 23, 13, and 95 mm in January, February, and March, respectively (Figure 3). Only 1 month—that is, December 2011—showed a very minor CH_4_ uptake.

Averaged over the year and separately for the growing and non‐growing season, the plantation was a small net source of N_2_O, except during the non‐growing season of 2013, when the plantation was approximately N_2_O neutral. The emission of N_2_O remained low immediately following land conversion to SRC, but in August 2010 (5 months after planting), one intense emission peak of short duration was observed (maximum daily average of 5.43 CO_2_ eq µmol m^−2^ s^−1^ on August 21, 2010; Figure 3). This single emission peak was most probably linked to the largest rainfall event that occurred during the study period, when rainfalls of 56.4 and 52.5 mm occurred on August 15–16, 2010 (see Figure 3; see also Zona et al., 2013). The sudden emission peak contributed significantly to the overall net N_2_O release over the entire period. Small, but not negligible correlations were found between the average N_2_O emission and both the precipitation and the average water table depth at a daily resolution, but not at a monthly resolution (Figure S4a,b). We observed a high annual N_2_O emission in 2016 (1.5 Mg CO_2_ eq ha^−1^ year^−1^; Figure 1) due to a second emission peak between May 24 and June 9, 2016 (Figure 3). The yearly absolute COV was high for 2010 (3.29 because of the single large pulse); for the other years, it ranged between 1.26 (2012) and 1.97 (2013). In total, 51.2 Mg/ha of CO_2_ was captured in 7 years. CH_4_ and N_2_O emissions amounted to 8.9 and 6.5 Mg CO_2_ eq/ha, respectively, reducing the total GHG uptake.

## 4 DISCUSSION

### 4.1 From a net source to a net sink of GHGs

Although previous studies mentioned that bioenergy production from woody crops can be sustainable after land conversion (Gelfand et al., 2013; Whitaker et al., 2017), the present study provides a unique dataset (seven full years, three rotations) of the total GHG balance of SRC from the continuous on‐site monitoring of CO_2_, CH_4_, and N_2_O fluxes over several rotations. Overall, the plantation became a net GHG sink after 5 years of SRC culture, confirming our hypothesis and previous studies. An extensive review of 138 studies showed that a decrease in GHG emissions occurs after land conversion to a (perennial or annual) bioenergy cropping system, with 10 years (on average) being needed to overrule the surplus in GHG emissions caused by the land conversion itself (Harris et al., 2015). At our site, the absence of fertilization may have led to the relatively short period needed to compensate this short‐term GHG emission surplus. Generally, after conversion of agricultural land to poplar SRC, no fertilization is needed for some 20 years. This is due to the effective recycling of leaf litter (Meiresonne, Schrijver, & Vos, 2007) and the low nutrient demand of poplar (Balasus, Bischoff, Schwarz, Scholz, & Kern, 2012), although the length of time depends on former land use, soil type, and site management. Also, because of the absence of annual soil tillage under SRC as in the case of annual crops, the CO_2_ emissions due to soil disturbance are minimized. The heavy machinery used during harvest may also lead to soil compaction with less aeration and water infiltration affecting the biological processes and related GHG effluxes from the soil (Epron et al., 2016; Sabbatini et al., 2016). The effect of a larger rooting system could cancel out this effect, depending on the poplar genotype (Berhongaray, Janssens, King, & Ceulemans, 2013) and the soil.

### 4.2 Non‐CO_2_ fluxes

The conversion of agricultural land into SRC can induce short‐term peaks in N_2_O emission (Nikiema et al., 2012; Palmer et al., 2014; Walter et al., 2015; Zona et al., 2012), comparable to the effect of tillage on precultivation soils leading to a rapid destabilization of the carbon and nitrogen cycles (Grandy & Robertson, 2006). Depending on the availability of ${\text{NO}}_{3}^{-}$ and water during the period following land conversion, the peak emission can appear immediately or may lag behind (Pinto et al., 2004). At our plantation, the peak N_2_O emission was important (39% of the annual net GHG emissions) during the establishment phase (August 2010) of the SRC. Before August 2010, plants were too small for a significant uptake of nitrogen, and thus, more nitrogen became progressively available for leaching and for microbial processes as a result of the aerobic nitrification of ${\text{NH}}_{4}^{+}$ to ${\text{NO}}_{2}^{-}$ and ${\text{NO}}_{3}^{-}$. Subsequently, the high rainfall in mid‐August 2010 probably created ideal conditions for the anaerobic denitrification of ${\text{NO}}_{3}^{-}$, causing the sudden production and subsequent emission of N_2_O. For six sites in the Northern Lake States of the United States, ${\text{NO}}_{3}^{-}$ availability explained 72% of the variation in the cumulative N_2_O flux (Palmer et al., 2014). Soil water content, soil temperature, and soil pH (when pH is low, the reduction of N_2_O to N_2_ is lower) are also important drivers of temporal variation in N_2_O (Walter et al., 2015), together with soil type and site management (Palmer et al., 2014). The stock of nitrogen in the soil of our site was high at the moment of establishment, that is, 9.4 ± 1.4 and 9.1 ± 2.1 Mg N/ha for the previous pasture and cropland, respectively (Broeckx et al., 2012) as a result of the long history of intensive crop production and the high nitrogen depositions in Flanders (Verstraeten et al., 2012).

In contrast to previous studies that showed a minor uptake of CH_4_ by SRC plantations (Drewer, Finch, Lloyd, Baggs, & Skiba, 2012; Gauder, Butterbach‐Bahl, Graeff‐Hönninger, Claupein, & Wiegel, 2012; Walter et al., 2015), our site was never a CH_4_ sink, but almost always a small source. This might most probably be explained by the high soil wetness creating anaerobic conditions, but which also favored the fast growth and the high yields of the SRC (Vanbeveren & Ceulemans, 2018; Verlinden et al., 2015). Emission events were nearly always related to short (1–2 days) periods of flooding and water logging at some parts of the plantation after intensive precipitation as confirmed by the low water table depths. Soil texture including clay‐enriched deeper soil layers (Broeckx et al., 2012) might also have contributed. Base CH_4_ emission fluxes—beside the emission events—were close to the detection limit of the analyzer. There was no short‐term effect of land conversion to SRC on the CH_4_ fluxes in line with previous observations at two sites in Germany (Walter et al., 2015).

### 4.3 Drivers of temporal and inter‐annual dynamics

Our study confirmed that our SRC in Flanders, Belgium, mitigated GHG emission a few years after establishment, that is, after two rotations. CO_2_ most strongly determined the overall GHG balance and the sink became larger with time. The conversion from agricultural land to SRC and the subsequent coppicing were the main causes of the emission of CO_2_, as well as of N_2_O (cf. Whitaker et al., 2017). We were not able to identify unique controlling factors of the temporal variation and the longer term evolution of CO_2_ and non‐CO_2_ fluxes (see Figures S2–S4). Furthermore, our sets of ancillary, explanatory data did not cover the entire 2010–2016 time period and, thus, our data analyses did not allow to unambiguously identify the drivers of the temporal and inter‐annual dynamics of the CO_2_ and of the GHG balance. The results of previous intensive campaigns and field observations between 2010 and 2016 suggest, however, that the following might explain the increasing CO_2_ sink with time: (a) the root system kept on increasing over the years and the rotations. So, the below‐ground root system grew bigger each year, while the above‐ground foliage and shoots were removed with each coppice; (b) growth vigor and resprouting performance increased over the years and the rotations (Vanbeveren & Ceulemans, 2018); (c) leaf area index increased and increased fast over the years (Vanbeveren et al., 2016); (d) over the period 2010–2014, we measured an increase of SOC sequestration of 9 Mg C/ha or 33 Mg CO_2_/ha (Berhongaray et al., 2017). So, without any doubt, many drivers (climate, phenology, coppicing, below‐ground carbon) jointly explained the dynamics in CO_2_ fluxes within as well as between years and rotations.

The conclusions of this study are based on the non‐irrigated, non‐fertilized, and only occasionally flooded SRC plantation in Flanders with its specific environmental conditions (of soil characteristics, soil water content and fertility, air temperature, and precipitation). Nevertheless, they illustrate the potential of SRC plantations to mitigate GHGs. Management options to further optimize the mitigation potential of land conversion to SRC might include, among others, longer rotations (involving less machinery, less GHG emissions in the whole life cycle), irrigation (higher yields, higher CO_2_ uptake rates), or drainage (lower GHG emissions) depending on the soil water status.

## References

[gcbb12648-bib-0001] Arevalo, C. B. M. , Bhatti, J. S. , Chang, S. X. , & Sidders, D. (2011). Land use change effects on ecosystem carbon balance: From agricultural to hybrid poplar plantation. Agriculture, Ecosystems & Environment, 141, 342–349. 10.1016/j.agee.2011.03.013

[gcbb12648-bib-0002] Aubinet M., Vesala T., & Papale D. (Eds.). (2012). Eddy covariance: A practical guide to measurement and data analysis. Dordrecht, the Netherlands: Springer Atmospheric Sciences, 424 pp.

[gcbb12648-bib-0003] Balasus, A. , Bischoff, W.‐A. , Schwarz, A. , Scholz, V. , & Kern, J. (2012). Nitrogen fluxes during the initial stage of willows and poplars in short‐rotation coppices. Journal of Plant Nutrition and Soil Science, 175, 729–738. 10.1002/jpln.201100346

[gcbb12648-bib-0004] Berhongaray, G. , Janssens, I. A. , King, J. S. , & Ceulemans, R. (2013). Fine root biomass and turnover of two fast‐growing poplar genotypes in a short‐rotation coppice culture. Plant and Soil, 373, 269–283. 10.1007/s11104-013-1778-x 25834288 PMC4372833

[gcbb12648-bib-0005] Berhongaray, G. , Verlinden, M. S. , Broeckx, L. S. , Janssens, I. A. , & Ceulemans, R. (2017). Soil carbon and belowground carbon balance of a short‐rotation coppice: Assessment from three different approaches. Global Change Biology Bioenergy, 9, 299–313. 10.1111/gcbb.12369 28261329 PMC5310368

[gcbb12648-bib-0006] Broeckx, L. S. , Verlinden, M. S. , & Ceulemans, R. (2012). Establishment and two‐year growth of a bioenergy plantation with fast‐growing *Populus* trees in Flanders (Belgium): Effects of genotype and former land use. Biomass & Bioenergy, 42, 151–163. 10.1016/j.biombioe.2012.03.005

[gcbb12648-bib-0007] Carter, M. , Hauggaard‐Nielsen, H. , Heiske, S. , Jensen, M. , Thomsen, S. T. , Schmidt, J. E. , … Ambus, P. (2012). Consequences of field N_2_O emissions for the environmental sustainability of plant‐based biofuels produced within an organic farming system. Global Change Biology Bioenergy, 4, 435–452. 10.1111/j.1757-1707.2011.01132.x

[gcbb12648-bib-0008] Ceulemans, R. , Scarascia‐Mugnozza, G. , Wiard, B. M. , Braatne, J. H. , Hinckley, T. M. , Stettler, R. F. , … Heilman, P. E. (1992). Production physiology and morphology of *Populus* species and their hybrids grown under short rotation. I. Clonal comparisons of 4‐year growth and phenology. Canadian Journal of Forest Research, 22, 1937–1948. 10.1139/x92-253

[gcbb12648-bib-0009] Conrad, R. (1996). Soil microorganisms as controllers of atmospheric trace gases H‐2, CO, CH_4_, OCS, N_2_O, and NO. Microbiological Reviews, 60, 609–640. 8987358 10.1128/mr.60.4.609-640.1996 PMC239458

[gcbb12648-bib-0010] Crutzen, P. J. , Mosier, A. R. , Smith, K. A. , & Winiwarter, W. (2008). N_2_O release from agro‐biofuel production negates global warming reduction by replacing fossil fuels. Atmospheric Chemistry Physics Discussions, 8, 389–395. 10.5194/acp-8-389-2008

[gcbb12648-bib-0011] Di Matteo, G. , Sperandio, G. , & Verani, S. (2012). Field performance of poplar for bioenergy in southern Europe after two coppicing rotations: Effects of clone and planting density. iForest – Biogeosciences and Forestry, 5, 224–229. 10.3832/ifor0628-005

[gcbb12648-bib-0012] Díaz‐Pinés, E. , Molina‐Herrera, S. , Dannenmann, M. , Braun, J. , Haas, E. , Willibald, G. , … Butterbach‐Bahl, K. (2016). Nitrate leaching and soil nitrous oxide emissions diminish with time in a hybrid poplar short‐rotation coppice in Southern Germany. Global Change Biology Bioenergy, 9, 613–626. 10.1111/gcbb.12367

[gcbb12648-bib-0013] Don, A. , Osborne, B. , Hastings, A. , Skiba, U. , Carter, M. S. , Drewer, J. , … Zenone, T. (2012). Land‐use change to bioenergy production in Europe: Implications for the greenhouse gas balance and soil carbon. Global Change Biology Bioenergy, 4, 372–391. 10.1111/j.1757-1707.2011.01116.x

[gcbb12648-bib-0014] Drewer, J. , Finch, J. W. , Lloyd, C. R. , Baggs, E. M. , & Skiba, U. (2012). How do soil emissions of N_2_O, CH_4_ and CO_2_ from perennial bioenergy crops differ from arable annual crops? Global Change Biology Bioenergy, 4, 408–419. 10.1111/j.1757-1707.2011.01136.x

[gcbb12648-bib-0015] Epron, D. , Plain, C. , Ndiaye, F.‐K. , Bonnaud, P. , Pasquier, C. , & Ranger, J. (2016). Effects of compaction by heavy machine traffic on soil fluxes of methane and carbon dioxide in a temperate broadleaved forest. Forest Ecology and Management, 382, 1–9. 10.1016/j.foreco.2016.09.037

[gcbb12648-bib-0016] EU . (2018). Directive EU # 2018/2001 of the European Parliament and of the Council of 11 December 2018 on the promotion of the use of energy from renewable sources. Official Journal Luxembourg, 328, 82–209. http://data.europa.eu/eli/dir/2018/2001/oj

[gcbb12648-bib-0017] Field, J. L. , Marx, E. , Easter, M. , Adler, P. R. , & Paustian, K. (2016). Ecosystem model parameterization and adaptation for sustainable cellulosic biofuel landscape design. Global Change Biology Bioenergy, 8, 1106–1123. 10.1111/gcbb.12316

[gcbb12648-bib-0018] Forster, P. , Ramaswamy, V. , Artaxo, P. , Berntsen, T. , Betts, R. , Fahey, D. W. , … Van Dorland, R. (2007). Changes in atmospheric constituents and in radiative forcing In Solomon S., Qin D., Manning M., Marquis M., Averyt K., Tignor M. M. B., LeRoy Miller H., & Chen Z. (Eds.), Climate change 2007: The physical science basis. Contributions of working group I to the fourth assessment report of the Intergovernmental Panel on Climate Change (pp. 129–234). Cambridge: Cambridge University Press.

[gcbb12648-bib-0019] Gauder, M. , Butterbach‐Bahl, K. , Graeff‐Hönninger, S. , Claupein, W. , & Wiegel, R. (2012). Soil derived trace gas fluxes from different energy crops – Results from a field experiment in Southwest Germany. Global Change Biology Bioenergy, 4, 289–301. 10.1111/j.1757-1707.2011.01135.x

[gcbb12648-bib-0020] Gelfand, I. , Sahajpal, R. , Zhang, X. , Izaurralde, R. C. , Gross, K. L. , & Robertson, G. P. (2013). Sustainable bioenergy production from marginal lands in the US Midwest. Nature, 493, 514–517. 10.1038/nature11811 23334409

[gcbb12648-bib-0021] Grandy, A. S. , & Robertson, G. P. (2006). Initial cultivation of a temperate‐region soil immediately accelerates aggregate turnover and CO_2_ and N_2_O fluxes. Global Change Biology, 12, 1507–1520. 10.1111/j.1365-2486.2006.01166.x

[gcbb12648-bib-0022] Grigal, D. F. , & Berguson, W. E. (1998). Soil carbon changes associated with short‐rotation systems. Biomass & Bioenergy, 14, 371–377. 10.1016/S0961-9534(97)10073-3

[gcbb12648-bib-0023] Hansen, E. A. (1993). Soil carbon sequestration beneath hybrid poplar plantations in the North Central United States. Biomass & Bioenergy, 5, 431–436. 10.1016/0961-9534(93)90038-6

[gcbb12648-bib-0024] Harris, Z. M. , Spake, R. , & Taylor, G. (2015). Land use change to bioenergy: A meta‐analysis of soil carbon and GHG emissions. Biomass & Bioenergy, 82, 27–39. 10.1016/j.biombioe.2015.05.008

[gcbb12648-bib-0025] Labrecque, M. , & Teodorescu, T. I. (2005). Field performance and biomass production of 12 willow and poplar clones in short‐rotation coppice in Southern Quebec (Canada). Biomass & Bioenergy, 29, 1–9. 10.1016/j.biombioe.2004.12.004

[gcbb12648-bib-0026] Laureysens, I. , Bogaert, J. , Blust, R. , & Ceulemans, R. (2004). Biomass production of 17 poplar clones in a short rotation culture on a waste disposal site and its relation to soil characteristics. Forest Ecology and Management, 187, 295–309. 10.1016/j.foreco.2003.07.005

[gcbb12648-bib-0027] Ledin, S. , & Willebrand, A. (1996) Handbook on how to grow short rotation forests, Technical report, Swedish University of Agricultural Sciences, Uppsala, Sweden, 330 pp.

[gcbb12648-bib-0028] Liberloo, M. , Calfapietra, C. , Lukac, M. , Godbold, D. , Luo, Z.‐B. , Polle, A. , … Ceulemans, R. (2006). Woody biomass production during the second rotation of a bioenergy *Populus* plantation increases in a future high CO_2_ world. Global Change Biology, 12, 1094–1106. 10.1111/j.1365-2486.2006.01118.x

[gcbb12648-bib-0029] Meiresonne, L. , De Schrijver, A. , & De Vos, B. (2007). Nutrient cycling in a poplar plantation (*Populus trichocarpa* x *Populus deltoides* ‘Beaupre’) on former agricultural land in Northern Belgium. Canadian Journal of Forest Research, 37, 141–155. 10.1139/x06-205

[gcbb12648-bib-0030] Mishurov, M. , & Kiely, G. (2011). Gap‐filling techniques for the annual sums of nitrous oxide fluxes. Agricultural and Forest Meteorology, 151, 1763–1767. 10.1016/j.agrformet.2011.07.014

[gcbb12648-bib-0031] Nikiema, P. , Rothstein, D. E. , & Miller, R. O. (2012). Initial greenhouse gas emissions nitrogen leaching losses associated with converting pastureland to short rotation woody bioenergy crops in Northern Michigan. Biomass & Bioenergy, 39, 413–426. 10.1016/j.biombioe.2012.01.037

[gcbb12648-bib-0032] Njakou Djomo, S. , El Kasmioui, O. , De Groote, T. , Broeckx, L. S. , Verlinden, M. S. , Berhongaray, G. , … Ceulemans, R. (2013). Energy and climate benefits of bioelectricity from low‐input short rotation woody crops on agricultural land over a two‐year rotation. Applied Energy, 111, 1–9. 10.1016/j.apenergy.2013.05.017

[gcbb12648-bib-0033] Pacaldo, R. S. , Volk, T. A. , & Briggs, R. D. (2013). No significant differences in soil organic carbon contents along a chronosequence of shrub willow biomass crop fields. Biomass & Bioenergy, 58, 136–142. 10.1016/j.biombioe.2013.10.018

[gcbb12648-bib-0034] Palmer, M. M. , Forrester, J. A. , Rothstein, D. E. , & Mladenoff, D. J. (2014). Conversion of open lands to short rotation woody biomass crops: Site variability affects nitrogen cycling and N_2_O fluxes in the US Northern Lake States. Global Change Biology Bioenergy, 6, 450–464. 10.1111/gcbb.12069

[gcbb12648-bib-0035] Pinto, M. , Merino, P. , del Prado, A. , Estavillo, J. M. , Yamulki, S. , Gebauer, G. , … Oenema, O. (2004). Increased emissions of nitric oxide and nitrous oxide following tillage of a perennial pasture. Nutrient Cycling in Agroecosystems, 70, 13–22. 10.1023/B:FRES.0000049357.79307.23

[gcbb12648-bib-0036] Reichstein, M. , Falge, E. , Baldocchi, D. , Papale, D. , Aubinet, M. , Berbigier, P. , … Valentini, R. (2005). On the separation of net ecosystem exchange into assimilation and ecosystem respiration: Review and improved algorithm. Global Change Biology, 11, 1424–1439. 10.1111/j.1365-2486.2005.001002.x

[gcbb12648-bib-0037] Sabbatini, S. , Arriga, N. , Bertolini, T. , Castaldi, S. , Chiti, T. , Consalvo, C. , … Papale, D. (2016). Greenhouse gas balance of cropland conversion to bioenergy poplar short‐rotation coppice. Biogeosciences, 13, 95–113. 10.5194/bg-13-95-2016

[gcbb12648-bib-0038] Schweier, J. , Molina‐Herrera, S. , Ghirardo, A. , Grote, R. , Diaz‐Pines, E. , Kreuzwieser, J. , … Becker, G. (2017). Environmental impacts of bioenergy wood production from poplar short‐rotation coppice grown at a marginal agricultural site in Germany. Global Change Biology Bioenergy, 9, 1207–1221. 10.1111/gcbb.12423

[gcbb12648-bib-0039] Sixto, H. , Cañellas, I. , van Arendonk, J. , Ciria, P. , Camps, F. , Sánchez, M. , & Sánchez‐González, M. (2015). Growth potential of different species and genotypes for biomass production in short rotation in Mediterranean environments. Forest Ecology and Management, 354, 291–299. 10.1016/j.foreco.2015.05.038

[gcbb12648-bib-0040] Smith, P. (2004). Carbon sequestration in croplands: The potential in Europe and the global context. European Journal of Agronomy, 20, 229–236. 10.1016/j.eja.2003.08.002

[gcbb12648-bib-0041] US DOE . (2016). Billion‐Ton Report, jointly released by the U.S. Department of Energy and Oak Ridge National Laboratory (ORNL). https://www.energy.gov/eere/bioenergy/2016-billion-ton-report

[gcbb12648-bib-0042] Van de Walle, I. , Van Camp, N. , Van de Casteele, L. , Verheyen, K. , & Lemeur, R. (2007). Short‐rotation forestry of birch, maple, poplar and willow in Flanders (Belgium) I. Biomass production after 4 years of tree growth. Biomass & Bioenergy, 31, 267–275. 10.1016/j.biombioe.2007.01.019

[gcbb12648-bib-0043] Vanbeveren, S. P. P. , Bloemen, J. , Balzarolo, M. , Broeckx, L. S. , Sarzi‐Falchi, I. , Verlinden, M. S. , & Ceulemans, R. (2016) A comparative study of four approaches to assess phenology of Populus in a short‐rotation coppice culture. iForest – Biogeosciences and Forestry, 9, 682–689. 10.3832/ifor1800-009

[gcbb12648-bib-0044] Vanbeveren, S. P. P. , & Ceulemans, R. (2018). Genotypic differences in biomass production during three rotations of short‐rotation coppice. Biomass & Bioenergy, 119, 198–205. 10.1016/j.biombioe.2018.09.027

[gcbb12648-bib-0045] Verlinden, M. S. , Broeckx, L. S. , & Ceulemans, R. (2015). First vs. second rotation of a poplar short rotation coppice: Above‐ground biomass productivity and shoot dynamics. Biomass & Bioenergy, 73, 174–185. 10.1016/j.biombioe.2014.12.012

[gcbb12648-bib-0046] Verlinden, M. S. , Broeckx, L. S. , Wei, H. , & Ceulemans, R. (2013). Soil CO_2_ efflux after land use change to a bioenergy plantation with fast‐growing *Populus* trees – Influence of former land use, inter‐row spacing and genotype. Plant and Soil, 369, 631–644. 10.1007/s11104-013-1604-5 25834286 PMC4372829

[gcbb12648-bib-0047] Verstraeten, A. , Neirynck, J. , Genouw, G. , Cools, N. , Roskams, P. , & Hens, M. (2012). Impact of declining atmospheric deposition on forest soil solution chemistry in Flanders, Belgium. Atmospheric Environment, 62, 20–63. 10.1016/j.atmosenv.2012.08.017

[gcbb12648-bib-0048] Walter, K. , Don, A. , & Flessa, H. (2015). No general soil carbon sequestration under Central European short rotation coppices. Global Change Biology Bioenergy, 7, 727–740. 10.1111/gcbb.12177

[gcbb12648-bib-0049] Whitaker, J. , Field, J. L. , Bernacchi, C. J. , Cerri, C. E. P. , Ceulemans, R. , Davies, C. A. , … McNamara, N. P. (2017). Consensus, uncertainties and challenges for perennial bioenergy crops and land use. Global Change Biology Bioenergy, 10, 150–164. 10.1111/gcbb.12488 29497458 PMC5815384

[gcbb12648-bib-0050] Zenone, T. , Zona, D. , Gelfand, I. , Gielen, B. , Camino‐Serrano, M. , & Ceulemans, R. (2016). CO_2_ uptake is offset by CH_4_ and N_2_O emissions in a poplar short‐rotation coppice. Global Change Biology Bioenergy, 8, 524–538. 10.1111/gcbb.12269

[gcbb12648-bib-0051] Zona, D. , Janssens, I. A. , Aubinet, M. , Gioli, B. , Vicca, S. , Fichot, R. , & Ceulemans, R. (2013). Fluxes of the greenhouse gases (CO_2_, CH_4_ and N_2_O) above a short‐rotation poplar plantation after conversion from agricultural land. Agricultural and Forest Meteorology, 169, 100–110. 10.1016/j.agrformet.2012.10.008

[gcbb12648-bib-0052] Zona, D. , Janssens, I. A. , Gioli, B. , Jungkunst, H. F. , Serrano, M. C. , & Ceulemans, R. (2012). N_2_O fluxes of a bioenergy plantation during a two years rotation period. Global Change Biology Bioenergy, 5, 536–547. 10.1111/gcbb.12019

