# Supplementary material for: Greenhouse gas budget of a poplar bioenergy plantation in Belgium: CO2 uptake outweighs CH4 and N2O emissions
Source: Glob Change Biol Bioenergy. 2019 Oct 6;11(12):1435–43. doi: 10.1111/gcbb.12648 (PMC6919937; doi:10.1111/gcbb.12648)
Supplement: Supplementary file 1 [file GCBB-11-1435-s001.docx]

**Supplementary Materials**


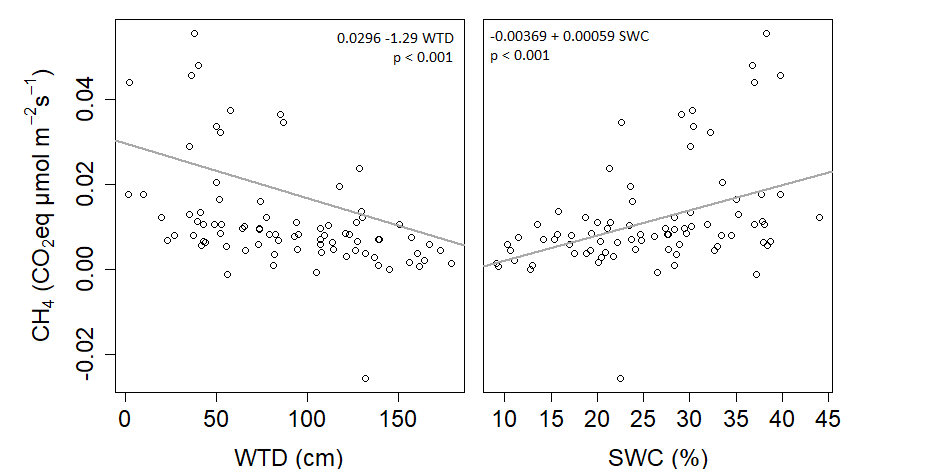


Figure S1: Correlation between the average monthly emission of CH_4_ expressed in CO_2_ equivalents (µmol m^-1^ s^-1^) and the average monthly water table depth (WTD, cm) and soil water content at 20 cm (vol%, m^3^ m^-3^).


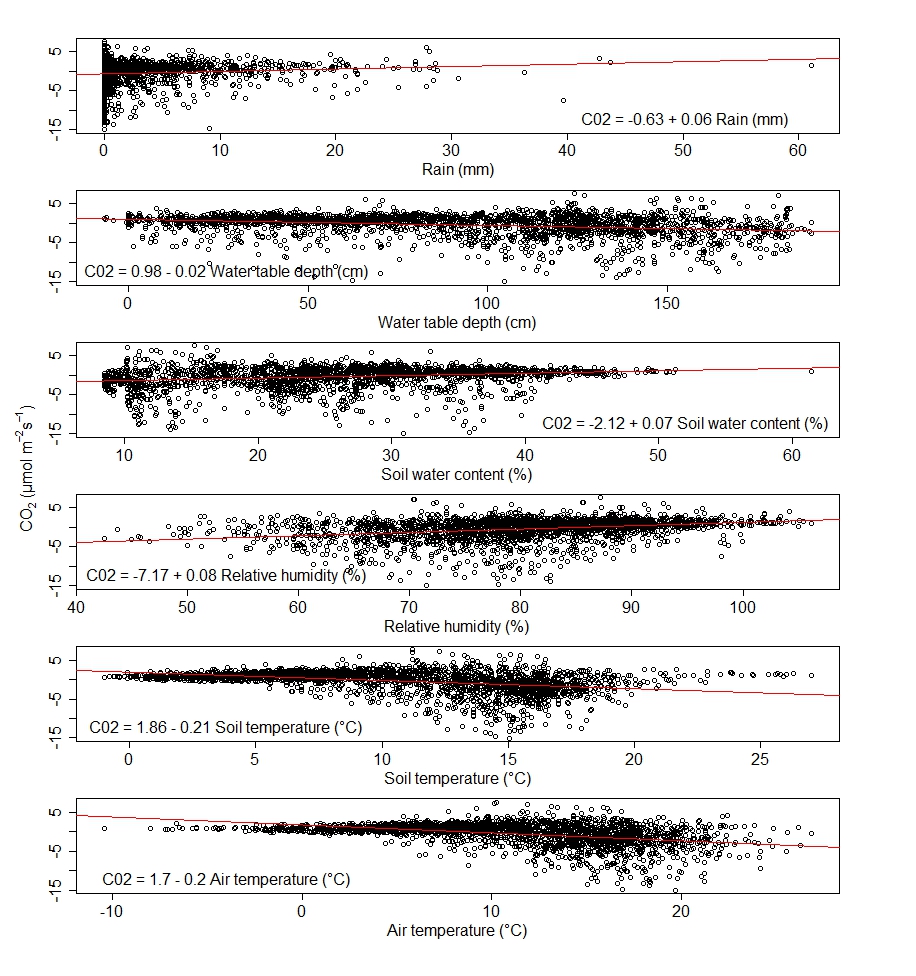


Figure S2A: Correlation between the average daily CO_2_ fluxes and the average daily precipitation, water table depth, soil water content (at 20 cm; vol%), relative humidity, soil temperature and air temperature.


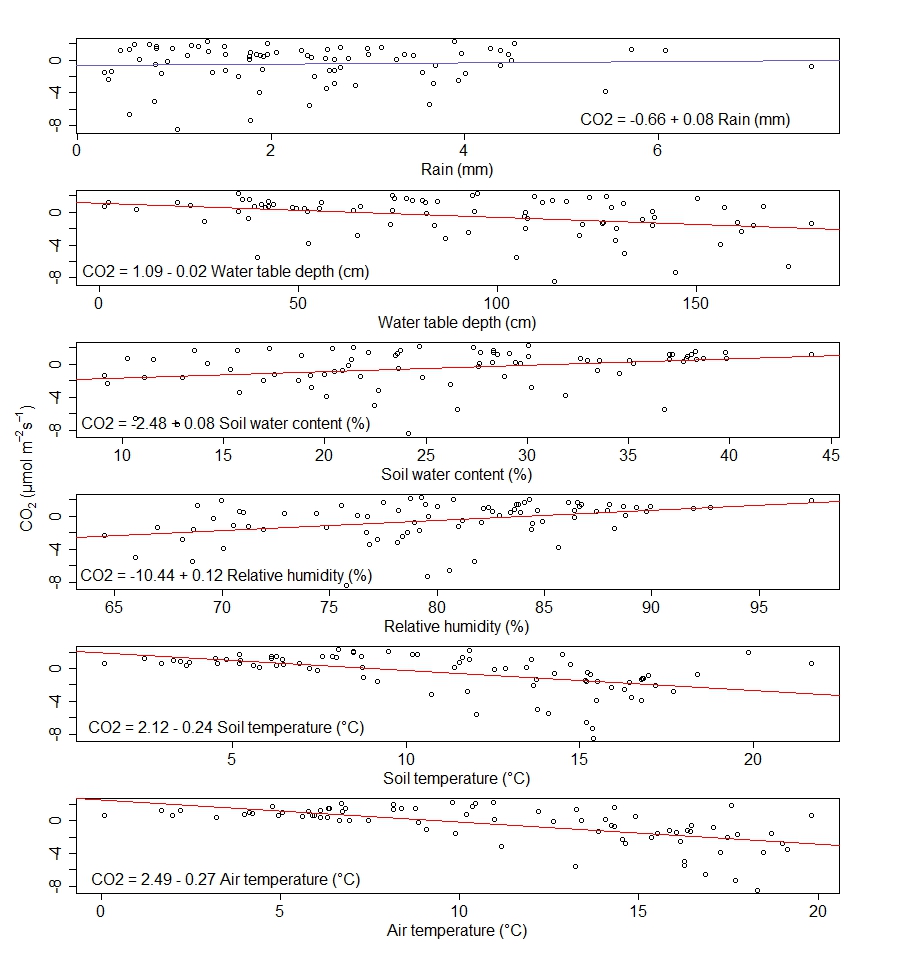


Figure S2B: Correlation between the average monthly CO_2_ fluxes and the average monthly precipitation, water table depth, soil water content (at 20 cm; vol%), relative humidity, soil temperature and air temperature.


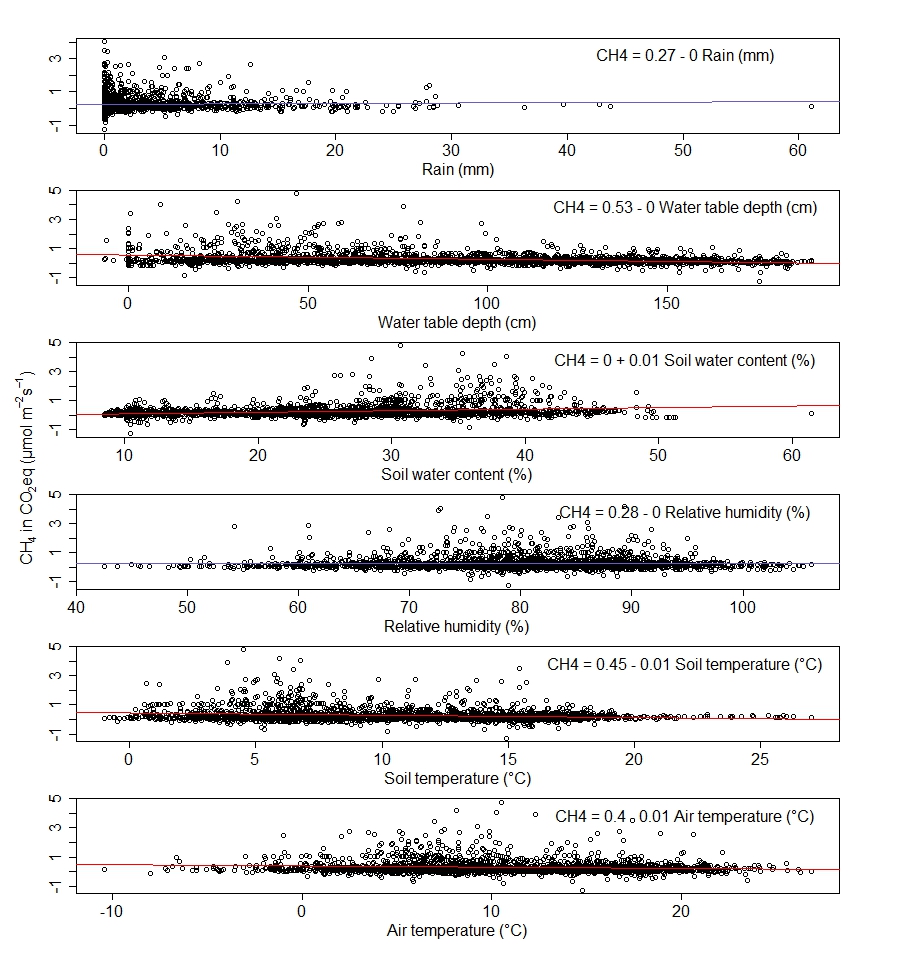


Figure S3A: Correlation between the average daily CH_4_ fluxes expressed in CO_2_ equivalents (µmol m^-1^ s^-1^) and the average daily precipitation, water table depth, soil water content (at 20 cm; vol%), relative humidity, soil temperature and air temperature.


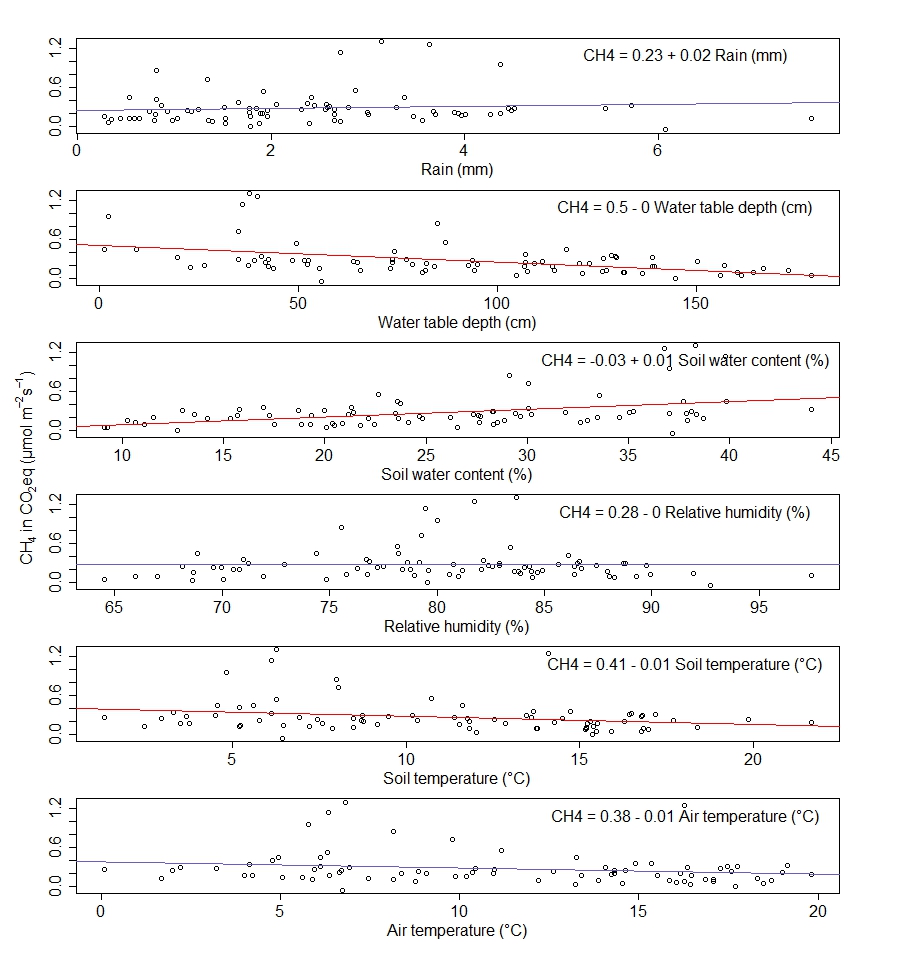


Figure S3B: Correlation between the average monthly CH_4_ fluxes expressed in CO_2_ equivalents (µmol m^-1^ s^-1^) and the average monthly precipitation, water table depth, soil water content (at 20 cm; vol%), relative humidity, soil temperature and air temperature. Water table depth explained 22% of the monthly variation of the fluxes . Soil water content at 0.2 m depth explained 16% of the monthly variation. Correlations between fluxes and both parameters were significant (respectively -0.47 and 0.41; p < 0.001).


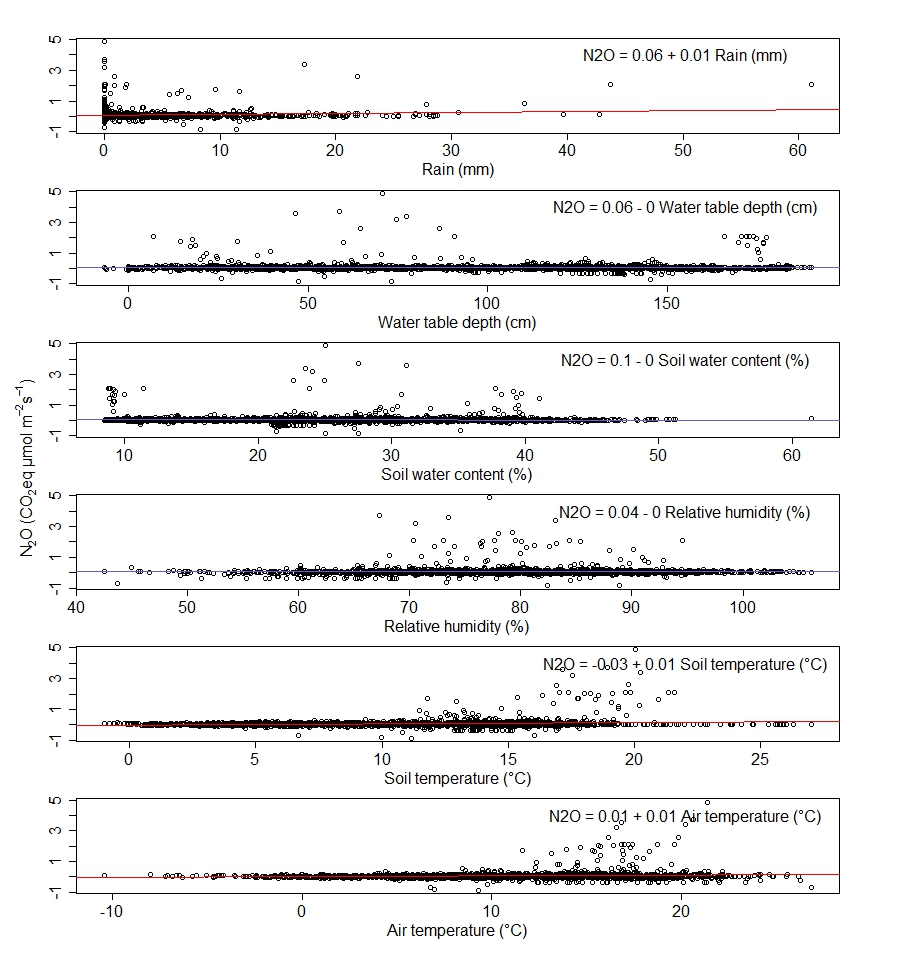


Figure S4A: Correlation between the average daily N_2_O fluxes expressed in CO_2_ equivalents (µmol m^-1^ s^-1^) and the average daily precipitation, water table depth, soil water content (at 20 cm; vol%), relative humidity, soil temperature and air temperature. Correlation coefficients between average N_2_O flux and precipitation (0.09; p < 0.001) and average water table depth (-0.04, p = 0.04) were significant.


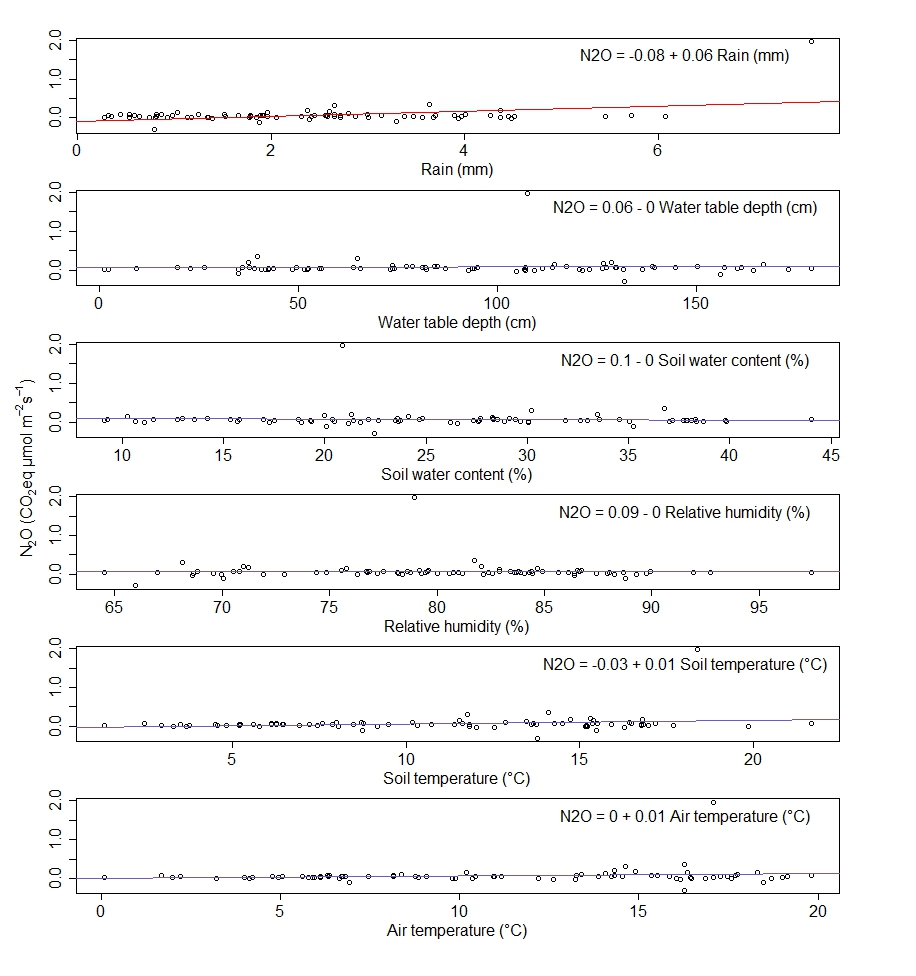


Figure S4B: Correlation between the average monthly N_2_O fluxes expressed in CO_2_ equivalents (µmol m^-1^ s^-1^) and the average monthly precipitation, water table depth, soil water content (at 20 cm; vol%), relative humidity, soil temperature and air temperature.
